# Preoperative prediction model for microvascular invasion in HBV-related intrahepatic cholangiocarcinoma

**DOI:** 10.1186/s12893-023-02139-8

**Published:** 2023-08-17

**Authors:** Liang Yu, Mu-Gen Dai, Wen-Feng Lu, Dong-Dong Wang, Tai-Wei Ye, Fei-Qi Xu, Si-Yu Liu, Lei Liang, Du-Jin Feng

**Affiliations:** 1 Department of Radiology, Cancer Center, Zhejiang Provincial People’s Hospital, Affiliated People’s Hospital, Hangzhou Medical College, Zhejiang Hangzhou, China; 2 Department of Gastroenterology, The Fifth Affiliated Hospital of Wenzhou Medical University, Lishui, Zhejiang China; 3 Department of Hepatobiliary Surgery, Eastern Hepatobiliary Surgery Hospital, Navy Medical University, Shanghai, China; 4 Department of Hepatobiliary & Pancreatic Surgery and Minimally Invasive Surgery , General Surgery, Cancer Center, Zhejiang Provincial People’s Hospital, Affiliated People’s Hospital, Hangzhou Medical College, Zhejiang Hangzhou, China; 5 Department of Laboratory Medicine, The Key Laboratory of Imaging Diagnosis and Minimally Invasive Interventional Research of Zhejiang Province, Zhejiang University Lishui Hospital, Lishui, Zhejiang China; 6 Department of Clinical Laboratory, Laboratory Medicine Center, Zhejiang Provincial People’s Hospital, Affiliated People’s Hospital, Hangzhou Medical College, Zhejiang 310014 Hangzhou, China; 7 Department of Laboratory Medicine Center, Zhejiang Center for Clinical Laboratories, Zhejiang Provincial People’s Hospital, Affiliated People’s Hospital, Hangzhou Medical College, Hangzhou, China

**Keywords:** Intrahepatic cholangiocarcinoma, Microvascular invasion, Prediction model, Hepatitis B virus, Preoperative

## Abstract

**Background and aims:**

Preoperative prediction of microvascular invasion (MVI) using a noninvasive method remain unresolved, especially in HBV-related in intrahepatic cholangiocarcinoma (ICC). This study aimed to build and validate a preoperative prediction model for MVI in HBV-related ICC.

**Methods:**

Patients with HBV-associated ICC undergoing curative surgical resection were identified. Univariate and multivariate logistic regression analyses were performed to determine the independent risk factors of MVI in the training cohort. Then, a prediction model was built by enrolling the independent risk factors. The predictive performance was validated by receiver operator characteristic curve (ROC) and calibration in the validation cohort.

**Results:**

Consecutive 626 patients were identified and randomly divided into the training (418, 67%) and validation (208, 33%) cohorts. Multivariate analysis showed that TBIL, CA19-9, tumor size, tumor number, and preoperative image lymph node metastasis were independently associated with MVI. Then, a model was built by enrolling former fiver risk factors. In the validation cohort, the performance of this model showed good calibration. The area under the curve was 0.874 (95% CI: 0.765–0.894) and 0.729 (95%CI: 0.706–0.751) in the training and validation cohort, respectively. Decision curve analysis showed an obvious net benefit from the model.

**Conclusion:**

Based on clinical data, an easy model was built for the preoperative prediction of MVI, which can assist clinicians in surgical decision-making and adjuvant therapy.

## Introduction

Intrahepatic cholangiocarcinoma (ICC) accounts for 5-30% of all primary liver cancers, and its incidence has been increasing in recent 30 years [1]. Surgical resection for the ICC remains the only potentially curative treatment but is associated with a high rate of tumor recurrence [2, 3]. How to improve preoperative surgical path planning and postoperative anti-recurrence treatment for ICC is a hot and difficult topic in clinical research.

Resection margin and microvascular invasion (MVI) are two important independent risk factors determining the poor prognosis of ICC undergoing surgical resection [4–6]. Previous studies have demonstrated that a wide resection margin (> 1 cm) can obviously improve overall survival and decrease the incidence of tumor recurrence [6]. Spolverato et al. indicated that with the decrease in the margin, the prognosis of patients was correspondingly worse [7]. Of note that, many patients with ICC are associated with hepatitis B virus (HBV) related cirrhosis, thus, the scope of surgical resection cannot be expanded at will [4, 8]. To ensure adequate residual liver volume, the surgeon is often forced to preserve more of the liver as possible. MVI refers to microscopically visible tumor infiltration in the hepatic vein, portal vein, or larger cystic blood vessels surrounding the liver tissue adjacent to the tumor, which is only visible under the microscope [5, 9]. Given that the incidence of residual tumor in the liver after liver resection, a wide margin resection was also required. Therefore, how to predict the presence of MVI before surgery is of great significance in guiding the surgical path planning and resection scope of liver. For example, in a patient with negative MVI, the surgeon can reserve more liver to reduce the risk of postoperative liver failure. However, preoperative prediction of MVI in ICC by noninvasive methods remains unresolved.

A multi-center retrospective study was conducted to build and validate a model for predicting MVI in HBV-related ICC patients before surgical resection. The purpose in the present study is to guide the surgical approach through preoperative prediction of MVI, so as to benefit patients and avoid more aggressive surgery.

## Patients and methods

### Patients

Patients with HBV-associated ICC after R0 resection were enrolled from Jan 2010 to Nov 2020 in the Zhejiang Provincial People’s Hospital in Hangzhou and the Eastern Hepatobiliary Surgery Hospital (EHBH) in Shanghai, China. All these patients were HBsAg -positive and did not receive anticancer treatment before surgery. The process of this study was guided by the Declaration of Helsinki and the Ethical Guidelines by the two hospitals. The Institutional Review Board of Zhejiang Provincial People’s Hospital and Eastern Hepatobiliary Surgery Hospital (EHBH) approved the study (No. QT2023181), and informed consent was obtained from all patients.

### Variables

The variables in the present study were retrospectively collected from the medical records system of Zhejiang Provincial People’s Hospital and EHBH [4]. The diagnosis of ICC is based on the pathologic results of the postoperative specimens [10]. The microscopic vascular invasion was defined as tumor invasion of intraparenchymal vascular identified on microscopy [11]. The patient-related and liver function-related variables included the age, sex, comorbid illnesses, total bilirubin (TBIL), preoperative serum albumin (ALB), alanine aminotransferase (ALT), aspartate transaminase (AST), gamma-glutamyl transpeptidase (GGT), prothrombin time (PT), platelet count (PLT) and cirrhosis. The cancer-related variables included preoperative carbohydrate antigen (CA) 19 − 9, carcinoma embryonic antigen (CEA), alpha-fetoprotein (AFP), tumor size and number. Preoperative image lymph node status was identified by imaging studies including contrast-enhanced CT, and/or MRI [12, 13]. To improve sensitivity, radiographically suspected lymph node metastases were classified into positive groups.

### Statistical analysis

In order to facilitate clinical application, continuous variables were stratified into binary categories. The cut-off value for continuity variable was according to previous related studies. Categorical variables were presented as number (n, %). Independent risk factors of MVI were identified by multivariable analyses. The independent risk factors were identified to construct a prediction model [14]. To evaluate fit of the prediction model, the performance was determined by discrimination and calibration. the area under the ROC curve (AUC) was used to evaluate the discrimination [15]. The calibration plot was evaluated by using the Hosmer–Lemeshow test. The discrimination and calibration also identified in the validation cohort. Decision curve analysis (DCA) was performed to assessed the predictive performance of the prediction model [16, 17]. All statistical analyses were conducted by the R 3.5.4 (http://www.r-project.org/). Statistical significance levels were set at *P* < 0.05.

## Results

### Baseline characteristics

Connective 626 patients received curative hepatectomy for HBV-related ICC. In the whole cohort, the overwhelming majority of patient were male (*n* = 468, 74.8%) and the median age was 54 years (range, 20 ~ 84 years). 544 (86.9%) patients were determined as lymph node metastasis by preoperative contrast-enhanced CT or MRI, including 92 (14.7%) patients with suspected lymph node metastasis. Meanwhile, 172 (27.5%) patients were diagnosed with cirrhosis by ultrasound, or contrast-enhanced CT or MRI. Among them, 115 (18.4) patients revived anatomical resection and 226 (36.2%) patients received larger resection (more than 3 hepatic segments). In addition, 107 (17.1%) patients were identified with MVI. Then, all 626 patients were randomly assigned into the training (418, 67%) and validation (208, 33%) cohorts for further analysis (Table 1).

**Table 1:** Clinical characteristics of the study population

N, %	The entire cohort (*N* = 626)	The training cohort (*N* = 418)	The validation cohort (*N* = 208)
Sex, Male/Female	468/158 (74.8/25.2)	308/110 (73.7/26.3)	160/48 (76.9/23.1)
Age, < 60 /≥60 years	419/207 (66.9/33.1)	282/136 (67.5/32.5)	137/71 (65.9/34.1)
Co-morbid illness, No/Yes	473/153 (75.6/24.4)	319/99 (75.4/24.6)	154/54 (74.0/26.0)
TBIL, ≤ 23 />23 µmol/L	477/149 (76.2/23.8)	364/115 (76.0/24.0)	162/46 (77.9/22.1)
ALB, ≤ 35 />35 g/L	565/61 (90.3/9.7)	376/42 (90.0/10.0)	189/19 (90.9/9.1)
ALT, ≤ 40 />40 IU/L	427/199 (68.2/31.8)	285/133 (68.2/31.8)	142/66 (68.3/31.7)
AST, ≤ 40 />40 U/L	489/140 (77.6/22.4)	319/99 (76.3/23.7)	142/66 (74.4/25.6)
GGT, ≤ 60 />60 U/L	293/333 (46.8/53.2)	196/222 (46.9/53.1)	97/111 (46.6/53.4)
PT, ≤ 13 />13 S	315/311 (50.3/49.7)	210/208 (50.2/49.8)	105/103 (50.5/49.5)
Platelet count, ≤ 100 />100 × 10^9^/L	567/59 (90.6/9.4)	376/42 (90.0/10.0)	191/17 (91.8/8.2)
AFP level, ≤ 20 />20 ug/L	466/160 (74.4/25.6)	314/104 (75.1/24.9)	152/56 (73.1/26.9)
CA19-9 level, ≤ 200 />200 U/mL	543/83 (86.7/13.3)	365/53 (87.3/12.7)	178/30 (85.6/14.4)
CEA level, ≤ 10 />10 ug/L	538/88 (85.9/14.1)	359/59 (85.9/14.1)	179/29 (86.1/13.9)
Maximum tumor size, ≤ 5 />5 cm	241/385 (38.5/61.5)	152/266 (36.4/63.6)	89/119 (42.8/57.2)
Tumor number, 1 /≥2	528/98 (84.3/15.7)	358/60 (85.6/14.4)	170/38 (81.7/18.3)
Preoperative image lymph node metastasis, No/Yes	544/82 (86.9/13.1)	368/50 (88.0/12.0)	176/32 (84.6/15.4)
Cirrhosis, No/Yes	454/172 (72.5/27.5)	303/115 (72.5/27.5)	151/57 (72.6/27.4)
Type of resection, anatomical	115 (18.4)	73 (17.5)	42 (20.2)
Major hepatectomy, ≥ 3 segments	226 (36.2)	158 (37.9)	68 (35.2)
MVI, No/Yes	519/107 (82.9/17.1)	352/66 (84.2/15.8)	167/41 (80.3/19.7)

*TBIL* Total bilirubin, *ALB* Albumin, *ALT* Alanine aminotransferase, *AST* Aspartate transaminase, *GGT* Gltamyltranspeptidase, *PT* Prothrombin time, *AFP* Alpha-fetoprotein, *MVI* Microscopic vascular invasion

### Independent risk factors of MVI

In the training cohort, the results by multivariable analysis showed that TBIL (OR 2.771, 95%CI 1.525–5.035, *P* < 0.001), CA19-9 (OR 2.095, 95%CI 1.004–4.370, *P* = 0.049), tumor size (OR 2.927, 95%CI 1.472–5.820, *P* < 0.001), tumor number (OR 2.661, 95%CI 1.370–5.168, *P* = 0.004), and preoperative image lymph node metastasis (OR 3.102, 1.536–6.265, *P* = 0.002) were independently associated with MVI (Table 2).

**Table 2:** Univariate and multivariate logistic regression analyses of preoperative variables in predicting microscopic vascular invasion in the training cohort

Variables	OR comparison	UV OR (95%CI)	UV *P*	MV OR (95CI)	MV *P*
Sex	Male vs. Female	1.393 (0.738–2.628)	0.306		
Age	≥ 60 /<60 years	1.232 (0.692–2.193)	0.479		
Co-morbid illness	Yes vs. No	0.797 (0.439–1.446)	0.456		
TBIL	> 23 vs. ≤23 µmol/L	2.321 (1.333–4.042)	0.003	2.771 (1.525–5.035)	< 0.001
ALB	≤ 35 vs. >35 g/L	1.786 (0.831–3.836)	0.137		
ALT level	> 40 vs. ≤40 IU/L	1.488 (0.864–2.536)	0.152		
AST level	> 40 vs. ≤40 IU/L	1.374 (0.763–2.475)	0.289		
GGT level	> 60 vs. ≤60U/L	1.955 (1.125–3.398)	0.017	NS	0.332
PT	> 13 vs. ≤13 S	1.817 (1.059–3.116)	0.030	NS	0.657
Platelet count	> 100 vs. ≤100 × 10^9^/L	1.075 (0.456–2.534)	0.869		
AFP level	> 20 vs. ≤20 ug/L	1.270 (0.707–2.282)	0.424		
CA19-9 level	> 200 vs. ≤200 U/mL	2.430 (1.247–4.735)	0.009	2.095 (1.004–4.370)	0.049
CEA level	> 10 vs. ≤10ug/L	2.300 (1.204–4.392)	0.012	NS	0.392
Maximum tumor size	> 5 vs.≤5 cm	2.972 (1.535–5.755)	0.001	2.927 (1.472–5.820)	< 0.001
Tumor number	≥ 2 vs. 1	3.391 (1.825–6.302)	< 0.001	2.661 (1.370–5.168)	0.004
Preoperative image lymph node metastasis	Yes vs. No	3.750 (1.953-7.200)	< 0.001	3.102 (1.536–6.265)	0.002
Cirrhosis	Yes vs. No	1.281 (0.725–2.263)	0.394		

*TBIL* Total bilirubin, *ALB* Albumin, *ALT* Alanine aminotransferase, *AST* Aspartate transaminase, *GGT* Gltamyltranspeptidase, *PT* Prothrombin time, *AFP* Alpha-fetoprotein, *MVI* Microscopic vascular invasion, *OR* Oddis ratio, *UV* Univariate, *MV* Multivariable, *NS* No significance

### Construction of the prediction model

**Fig. 1:**
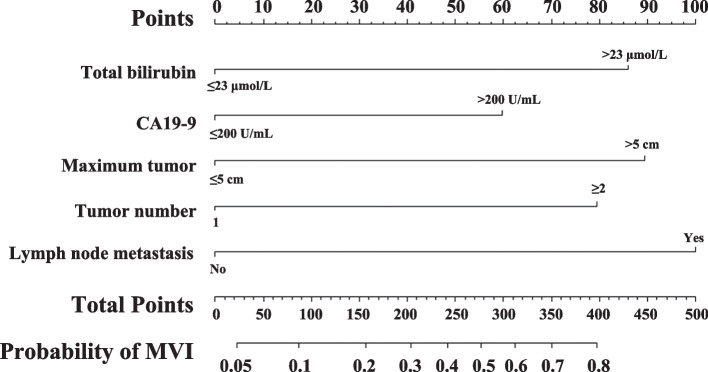
The nomogram model for the preoperative prediction of microvascular invasion in HBV-related intrahepatic cholangiocarcinoma

A nomogram models that integrated the five independent risk factors associated with MVI were constructed to predict MVI among patients with HBV-related ICC (Fig. 1). Each variable has a score. The estimated probability of MVI can be obtained by adding up all the scores, locating the total score on the total score scale and drawing a straight line vertically down.

### Validation of the prediction model

**Fig. 2:**
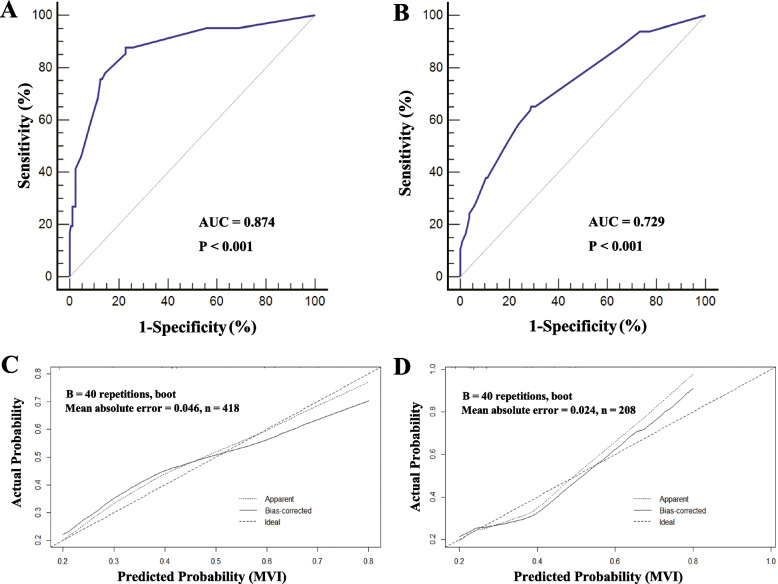
Receiver operating characteristic curves (**A**, in the training cohort, and **B**, in the validation cohort) and calibration plots (**C**, in the training cohort, and **D**, in the validation cohort) of the model for the prediction of microvascular invasion in HBV-related intrahepatic cholangiocarcinoma. The calibration plot compares the predicted and actual outcomes. The dashed line is a reference line, indicating where an ideal nomogram would be. The solid line indicates the 40-sample bootstrapped performance of the nomogram. The calibration plots lay close to the dashed lines when plotting the predicted probabilities against the actual probabilities, demonstrating that the calibration plots of the nomogram fitted well in both two cohorts. AUC, Area under the curve; CI, Confidence interval

The AUC was 0.874 (95% CI: 0.765–0.894) and 0.729 (95%CI: 0.706–0.751) in the training and validation cohort, respectively (Fig. 2A and B). The results demonstrated the prediction model with a good accuracy to estimate the probability of MVI. The calibration plots also showed a good fit in the training and validation cohort, which means a good agreement between the actual observation and the prediction for the probability of MVI (Fig. 2C and D).

### Performance of the prediction model

**Fig. 3:**
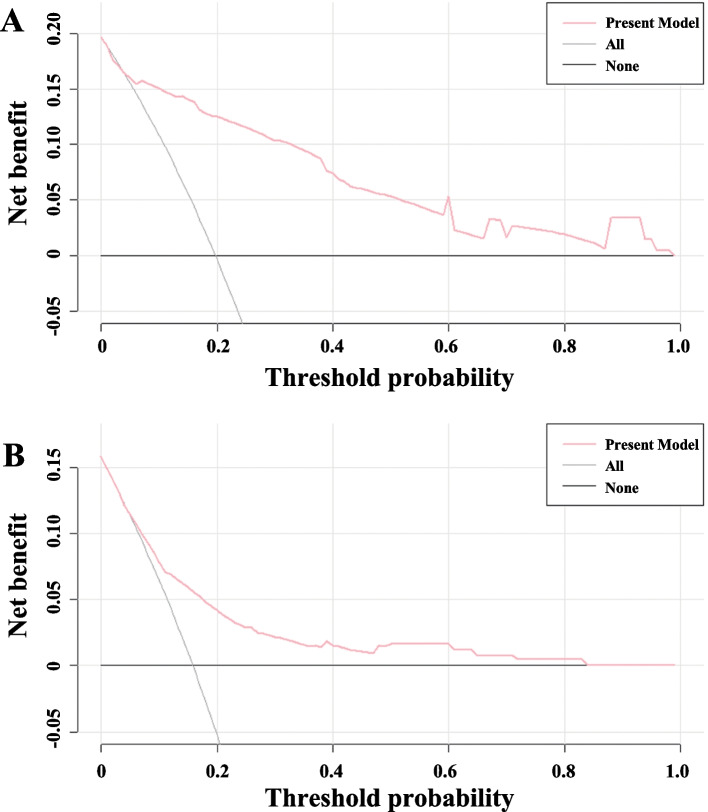
Decision curve analysis for the present model in the training cohort (**A**) and the validation cohort (**B**). The black line represents the assumption that the prediction of MVI was wrong in all patients. The Grey line represents the assumption that the prediction of MVI was right in all patients. The net benefit was weighted by the relative harm of the wrong prediction for MVI negative patients compared with the wrong prediction for MVI positive patients. Threshold probability is where the expected net benefit of the right prediction is equal to the expected net benefit of the false prediction

The optimal cut-off value for the prediction model nearly was 140 to distinguish the presence or absence of MVI. At the cut-off value, the specificity, sensitivity, negative and positive predictive value were 77.25% 87.80%, 96.30% and 48.60% in the training cohort, and 71.02%, 65.15%, 91.60% and 29.70% in the validation cohort (Table 3). Decision curve analysis showed a good net benefit in prediction of MVI both in the training and validation cohort (Fig. 3A and B).

**Table 3:** Performance indexes for the nomogram prediction model

Performance index	Training cohort	Validation cohort
Area under ROC curve	**0.874**	**0.729**
Cut-off score	142	139
R^2^	0.470	0.281
Brier scores	0.098	0.115
Specificity, %	77.25	71.02
Sensitivity, %	87.80	65.15
Negative predictive value, %	96.30	91.60
Positive predictive value, %	48.60	29.70
Negative likelihood ratio	0.16	0.49
Positive likelihood ratio	3.86	2.25

## Discussion

In the present study, a preoperative prediction model of MVI in HBV-related ICC was built and validated based on 626 patients undergoing curative surgical resection. Five independent risk factors, including TBIL, CA19-9, tumor size, tumor number, and preoperative image lymph node metastasis, were identified to constructed the prediction model. The discrimination showed the area under the ROC curve was 0.874 and 0.729 in the training and validation cohort, respectively. The calibration plots also showed a good fit, which means a good agreement between the actual observation and the prediction for the probability of MVI. Moreover, decision curve analysis showed a good net benefit from this preoperative prediction model of MVI. To our knowledge, this is the first preoperative prediction model of MVI for HBV-related ICC.

MVI refers to microscopically visible tumor infiltration in the portal vein, hepatic vein, or larger cystic blood vessels surrounding the liver tissue adjacent to the tumor, which is only visible under the microscope. MVI has been recognized as a clear and independent risk factor associated with tumor recurrence and overall survival after ICC curative resection, and is attracting increasing attention from the surgeons, pathologists, and researchers around the world [5, 18]. Previous studies also demonstrated that a wide resection margin can significantly decrease the tumor recurrence and increase the overall survival, when compared with a narrow resection margin [4, 6, 7, 19]. Lu et al. performed a retrospective study to evaluate the synergistic impact of resection margin and MVI for patients with HBV-related ICC. The results showed that a narrow resection margin with MVI is the greatest independent risk factor [7]. Of note that, many patients with ICC are associated with HBV related cirrhosis, thus, the scope of surgical resection cannot be expanded at will [4, 8]. To ensure adequate residual liver volume, the surgeon is often forced to preserve more of the liver as possible. In the present study, there are also 172 (27.5%) patients were diagnosed with cirrhosis. Thus, preoperative prediction of MVI is particularly important. However, preoperative assessment of MVI in ICC by using a noninvasive method is still an unresolved issue. Continued efforts to accurately predict MVI are important to counsel patients and guide treatment decisions. The model of predicting MVI in the present study showed a good discrimination and calibration by enrolling five preoperative variables.

We also noticed that there are mainly two published studies referring prediction MVI in ICC patients preoperatively [20, 21]. Chen et al. performed a multicenter study to prediction MVI in patients with ICC. The results showed that age, tumor number, and GGT were risk for the MVI [20]. Different from this study, the present study added the preoperative image lymph node status, which is significantly associated with MVI. Moreover, we only studied HBV-related ICC patients because we believe that these patients often have cirrhosis, which can have a significant impact on surgical treatment decisions. Another study by Ma et al. performed a prediction MVI model based on MRI image, including T1WI, T2WI, DWI, and dynamic enhancement imaging [21]. However, only 108 patients were enrolled in the study and no specific analysis of patients with HBV was performed. At the same time, we believe that image scoring is too dependent on senior imaging doctors, which is not conducive to the implementation of clinical application. The results of present study showed the prediction model good predictive power and clinical utility.

However, there are still some limitations in the present study. First, there is an inherent bias in retrospective studies. Thus, more validation in other centers and randomized controlled trial are still needed. Secondly, the present study only included HBV-related ICC. The prediction model needs to be validated in other patients with ICC. Thirdly, the accuracy of preoperative lymph nodes status based on image remains to be confirmed. We have noticed that several studies have explored this question [22–25]. However, more high-quality studies are still required.

## Conclusion

Based on clinical data, an easy model was built for the preoperative prediction of MVI, which can assist clinicians in surgical decision-making and adjuvant therapy.

## Data Availability

The datasets used and/or analysed during the current study available from the corresponding author on reasonable request.

## Abbreviations

MVI: Microvascular invasion
ICC: Intrahepatic cholangiocarcinoma
ROC: Receiver operator characteristic
HBV: Hepatitis B virus
AUC: Area under curve
PLT: Platelet count
HBV: Hepatitis B virus
CA19-9: Carbohydrate antigen 19 − 9
CEA: Carcinoma embryonic antigen; alpha-fetoprotein
MV: multivariable
NA: Not available
OR: Odds ratio
UV: Univariable
CI: Confidence interval
